# Skin thickness dimensions in histological section measurement during late‐fetal and neonatal developmental period: A systematic review

**DOI:** 10.1111/srt.12719

**Published:** 2019-05-22

**Authors:** Ingrid Michelle Fonseca de‐Souza, Gabriela Luiza Nogueira Vitral, Zilma Silveira Nogueira Reis

**Affiliations:** ^1^ Postgraduate Program in Women’s Health, Faculty of Medicine Universidade Federal de Minas Gerais Belo Horizonte Brazil; ^2^ Postgraduate Program in Pediatrics and Adolescence, Faculty of Medicine Universidade Federal de Minas Gerais Belo Horizonte Brazil

**Keywords:** barrier function, gestational age, histological techniques, review, skin, skin maturation, skin structure, systematic

## Abstract

**Background:**

The development and maturation of the skin is a process that occurs during the gestation and neonatal period. Histological skin biopsy studies are relevant to improve knowledge on the skin protective barrier during the perinatal period. The thin skin of preterm newborns is unable to maintain homeostasis, thermal regulation through the skin, and is susceptible to infections. This study systematically reviewed the evidence regarding histological thickness dimensions of the skin and its layers during the late‐fetal and neonatal period.

**Methods:**

PubMed, Scopus, BVS, and e SciELO library databases**,** with no limits in the period of analysis or idiom. Eligibility criteria were as follows: studies describing the thickness of the entire skin or its layers during late‐fetal life or the neonatal period; human being; skin biopsy analysis; and any scientific report. Two independent reviewers screened the search and extracted the following standard data: fetal or neonatal age of assessment, biopsy site, technique used for preparation and staining of histological slides, measurement techniques, and values of skin thickness.

**Results:**

Fifty‐nine studies were screened, and eleven were identified from other sources. We recognized six studies that met the criteria for inclusion for proper extraction. Expressive differences between sites for sampling, methods of slide preparation, and number of layers measured made the thicknesses values summarization difficult. There were no reliable dimensions reported on this tissue.

**Conclusion:**

Despite the importance of studying the human skin barrier, these findings confirmed limited evidence on skin thickness dimensions obtained by histology.

## 1 INTRODUCTION

The skin is considered the largest organ of the human body, having several vital functions^1^ and acting as a defensive physical barrier between the organism and the environment.^2^ The skin collaborates with other organs, providing consonant functioning of the organism, as well as control of body temperature and metabolic synthesis. Such relevance explains why the structural development of the human skin has been intensely studied and documented at the electron microscopy level.^3^ This tissue consists of dermis and epidermis, acting harmonically and cooperatively. The epidermal layer has a barrier function where the stratum corneum is positioned as having the outermost exposure to the environment.^1^

There are critical clinical relations between the skin barrier competence and the neonatal survival due to hypothermia and neonatal infections, besides risk factors for newborn deaths.^4^ The functional and structural development of the skin is a dynamic process, which begins during embryogenesis and ends in the first year of life.^1^, ^5^ Even the barrier maturation has particular importance during the late gestation and early neonatal period, histological studies are limited due to its invasive acquisition of materials from the human being, for ethical issues.^3^ Microscopy analysis of the tissue suggests that the skin structure is complete at 34 weeks of pregnancy. Thus, term newborns already have a competent barrier, comparable to adults.^6^ In contrast, preterm neonates are poorly prepared to face the extrauterine environment, as they lack development in the epidermal skin layer.^6^ Such weakness on the immature stratum corneum increases susceptibility to infections and percutaneous uptake of harmful toxins, and also leading to inability to maintain homeostasis, poor thermoregulation, and more risk of death.^7^, ^8^

The measurement of skin thickness is an important parameter that indirectly reflects the state of neonatal maturity and how prepared the newborn will be in the period of adaptation to the external environment.^9^ The depth of this tissue and the structure of epidermal and dermal layers differ according to the body site analyzed.^10^ Over the palm, sole and along joints, the epidermal layer is thicker than other parts, whereas between scapulae, the dermal layer is thicker than others sites.^10^ In other sites of the fetal body, especially the eyelids and near the genitals, the skin is typically thin, since there is no lucidum sub‐layer in the epidermis and the stratum corneum is reduced.^10^, ^11^

A considerable part of the knowledge on skin morphophysiology relies on the mouse model for skin maturation analysis, experimental culture models, and recently noninvasive approaches, due to restrictions on access to human fetal tissue.^3^ However, invasive biopsies of skin are still indispensable in situations where histology is the gold standard as a reference for the validation of imaging exams, as well as the potential to diagnose cutaneous pathologies.^12^ This study seeks to systematically review the published evidence in order to establish the magnitude of the dimension of human skin thickness and its layers during late‐fetal development and the neonatal period, assessed by biopsies and histological analysis.

## 2 METHODS

This systematic review was conducted in accordance with the PRISMA Statement.^13^ The authors previously prepared the review protocol using the application software StArt (Systematic Review System).^14^ The research question that guided the study was: What is the thickness of the human skin at birth, directly measured by histology? The main outcome was the value of skin thickness.

### 2.1 Search strategy and selection criteria

The search strategy was performed using the following keyword combination for the composition of PICO query:

(((((measurement[Title/Abstract] OR thickness[Title/Abstract] OR mophometry[Title/Abstract])) AND (biopsy[Title/Abstract] OR microscopy[Title/Abstract] OR slice[Title/Abstract] OR histology[Title/Abstract])) AND (dermis[Title/Abstract] OR epidermis[Title/Abstract] OR skin[Title/Abstract] OR “stratum corneum”[Title/Abstract] OR corium[Title/Abstract])) AND (child[Title/Abstract] OR fetus[Title/Abstract] OR infant*[Title/Abstract] OR neonate[Title/Abstract] OR newborn[Title/Abstract] OR stillbirth[Title/Abstract])) AND human*[Title/Abstract].

The authors conducted a comprehensive search for published evidence in the PubMed, Scopus, Virtual Health Library (BVS), and SciELO databases, with no date or language limits. There were no restrictions on the design of the studies. Additionally, other sources of evidence were consulted, such as the bibliography present in specialized books, dissertations, and theses.

### 2.2 Study selection

Two reviewers independently screened the search output to identify potentially relevant studies, analyzing only titles and abstracts using the following predetermined eligibility criteria:

1. Fetal or neonatal specimen;
2. Skin biopsy analysis;
3. Human being;
4. Measure the thickness of the skin or its layers;

We excluded studies reporting only noninvasive techniques for human skin thickness. Duplicates were discarded. Disagreements were resolved by consensus.

### 2.3 Data analysis

The selected publications were fully and independently read for extraction. A standard data extraction supported by software^14^ gathered the following variables: authors, year of publication, fetal or neonatal age, body site of biopsy, skin layer, technique used for preparation and staining of histological slides, methods of measurement, and mean, median, or range values of total skin thickness or the layers: stratum corneum, epidermis, and dermis. When data were missing or unclear, the original authors were contacted by electronic mail to clarify critical points before the aggregation of skin thickness dimensions.

The summary of the primary outcome with values of skin thickness was organized by body site where biopsies occurred, whether it was measured during late‐fetal or neonatal period, and the skin layers measured in the primary studies. For standardization, in the case of multiple sites, the following data were extracted: sole, palm, abdomen, back, and capillitium. The original descriptive values in terms of mean, maximum, minimum, and standard deviation, when present, were recovered from the primary source. To facilitate the comparability of the measured values, the unit of measure was micrometer (μm). We performed transformation when necessary. When skin thickness was indirectly described, as using a linear regression equation along timing, the birth value was obtained by assuming age zero days in the prediction equation. For studies that evaluated the thickness of the fetal skin at different moments of fetal life, only the measurements assessed near the gestational term were extracted.

## 3 RESULTS

**Figure 1:**
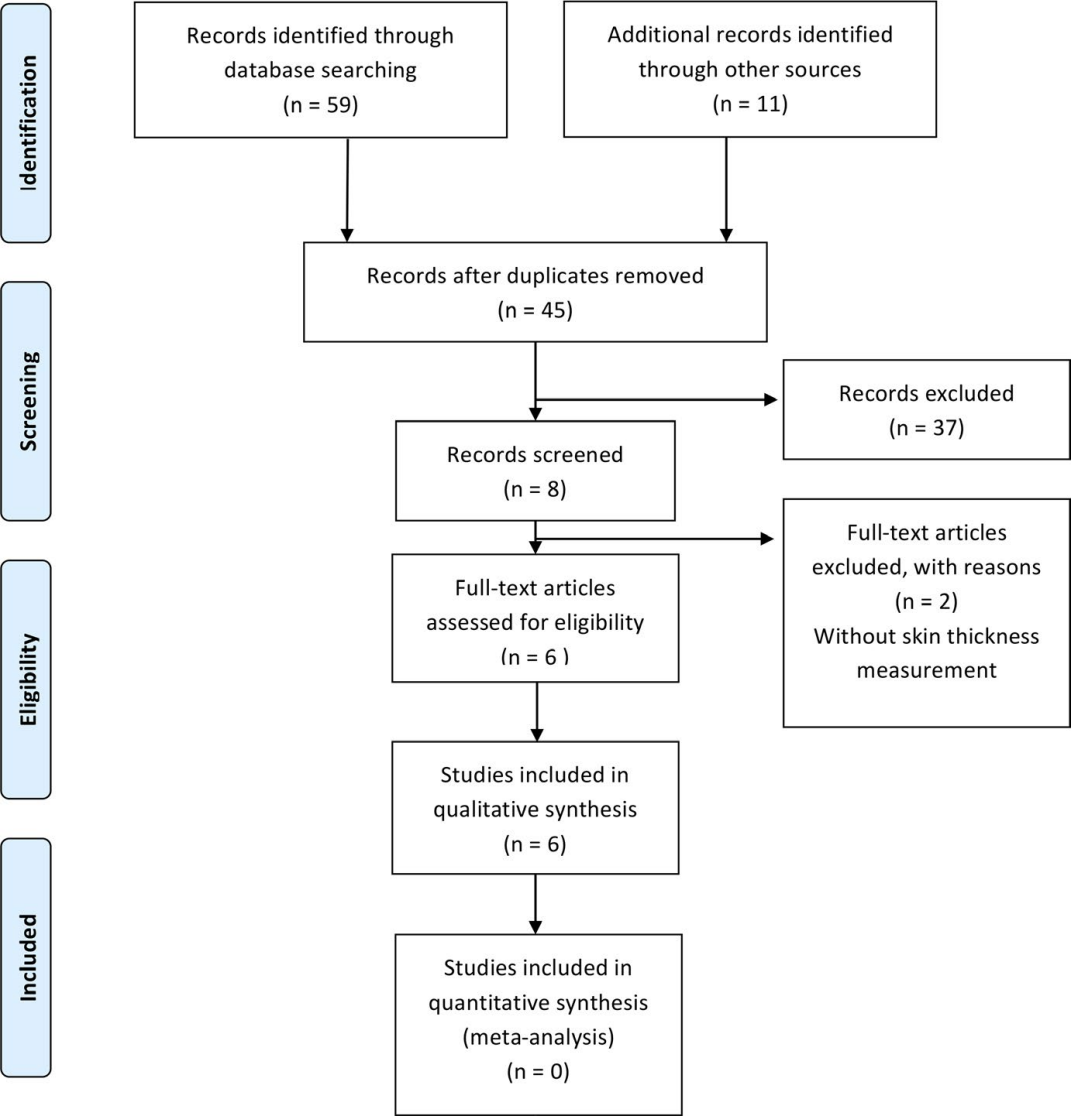
Flow diagram of systematic review [Colour figure can be viewed at http://wileyonlinelibrary.com]

Figure 1 illustrates the flow of identification, selection, and inclusion of studies, according to the PRISMA^13^ diagram. Bibliographic searching retrieved a total of 59 articles. We manually added eleven studies retrieved from specialized books and articles cited therein. Among the 70 selected, 25 duplicates were removed, resulting in 45 articles. Only eight primary sources met the eligibility criteria, and six reached the extraction step. Table S1 presents excluded articles as supplementary data to this article. Reasons for excluding potentially relevant studies was an approach of fetal or neonatal skin analysis without reporting skin thickness measurements.

The articles differed with dates of publication ranging from 1982 to 2018. Regarding quality of evidence, in coherence with our research question, only descriptive observational studies were assessed. Table 1 summarizes sample and histological techniques reported for the preparation of the slides from skin biopsies.

**Table 1:** Study characteristics and histological methods of sample preparation and skin thickness measurement

N	Authors	Sample of newborns	Methods of preparation	Layers analyzed	Method of measurements
1	Smith et al^15^	Unspecified number of fetuses	Biopsy dimensions: not reported. Fixation: immersed in 1/2 strength Karnovsky's fixative buffered in 0.1 mol/L cacodylate buffer and fixed for 2‐4 h. Samples were washed in buffer, and subsequently fixed in 1% OS04. Paraffin sections: 1 µm Coloration: Richardson, Jarrett, and Finke.	Dermis	Electron microscope and Philips 201 transmission electron microscope. Descriptive statistics of the values were not reported.
2.	Fairley et al^16^	10 infants children < 3 mo of age (autopsy)	Biopsy dimensions: not reported. Fixation: 10% buffered formalin. Paraffin sections: not reported. Coloration: HE.	Stratum corneum	Microscope with filar micrometer eyepiece calibrated for x10. Five measurements were made on each sample. Average with range values.
3.	De Viragh et al^17^	7 alive newborns after 2 wk of age, under anesthesia	Biopsy dimensions: not reported. Fixation: immediately after excision in Teller Nitzky solution (2% formaldehyde, 5% acetic acid, 65% ethanol) for exactly 24 h at room temperature. Paraffin sections: not reported Coloration: HE.	Epiderm (without stratum corneum) Derm (without adventitial layer)	Microscope with ocular micrometer. Minimum and maximum thickness for dermis and epidermis were measured. Average values with standard error, using regression analysis.
4.	Kakasheva‐Mazhenkovska et al^18^	Unspecified number of subjects. At least 10 samples of stillbirths at term gestation	Biopsy dimensions: 0.5‐1 cm Fixation: 10% neutral formalin. Paraffin sections: 3‐5 µm Coloration: HE, Azan—Mallory, PAS, Floranten, Linder technique of impregnation with silver.	Total epidermis Total skin Dermis	Computer system: Lucia M, Version 3, System for Image Processing and Analysis. Average without range values.
5.	Kakasheva‐Mazhenkovska et al^19^	Unspecified number of subjects. At least 12 samples of stillbirths at term gestation	Biopsy dimensions: 0.5 cm Fixation: not reported. Sections: not reported. Coloration: HE, Azan—Mallory, PAS, Floranten.	Total epidermis Total dermis	Computer system: Lucia M, Version 3, System for Image Processing and Analysis. Average without range values.
6.	Khalfa et al^10^	5 fetuses with 6 mo of age. 15 stillbirths	Biopsy dimensions: not reported. Fixation: optimum cutting temperature Freezing cryostat sections: 5 µm in −24°C. Coloration: HE. Scanning electron microscopy: Fixation: fresh tissues were embedded in glutaraldehyde and then processed with osmium tetroxide. Coloration: coated with gold	Epidermis	Scanning electron microscopy supported by software ImageJ. Average with range values.

Abbreviations: HE, hematoxylin and eosin; PAS, periodic acid–Schiff.

There was high variability between studies with respect to the sites of the body where the skin biopsy occurred, as well as methods for preparation and staining of slides. In addition, we observed an expressive diversity of techniques of measurement of skin thickness, microscopy equipment, and dedicated software for metering. Despite differing techniques of histological techniques of preparation, five of the six articles reported measurement supported by software. We contacted Khalfa et al^10^ to clarify the magnitude of skin thickness. The authors informed a mistake in their report regarding the unit of measure and sent us the corrected values, which we considered in our review.

The back was the body site whose skin thickness was most frequently measured, and five of six studies assessed the skin during the neonatal phase (Table 2).

**Table 2:** Skin thickness variation of newborns analyzed in the articles

	Study 1 Smith et al, 1982^15^	Study 2 Fairley et al, 1983^16^	Study 3 De Viragh et al, 1995^17^	Study 4 Kakasheva‐Mazhenkovska et al, 2011^18^	Study 5 Kakasheva‐Mazhenkovska et al, 2011^19^	Study 6 Khalfa et al, 2018^10^^,^ b	
Fetal	x					x	
Neonatal		x	x	x	x		x
Skin palm							
Stratum corneum (μm)	‐	‐		‐	‐	‐	‐
Total epidermis (μm)	‐	‐		142	‐	‐	‐
Dermis (μm)	‐	‐		873	‐	‐	‐
Total skin (μm)	‐	‐		1015	‐	‐	‐
Skin sole							
Stratum corneum (μm)	‐	‐		‐	‐		‐
Total epidermis (μm)	‐	‐		193.2	‐	470 ± 121	680 ± 315
Dermis (μm)	‐	‐		719.9	‐	‐	‐
Total skin (μm)	‐	‐		913. 1	‐	‐	‐
Skin abdomen							
Stratum corneum (μm)	‐	35.4 ± 11.3		‐	‐		‐
Total epidermis (μm)	‐	‐		161.6	‐	530 ± 111	650 ± 331
Dermis (μm)	‐	‐		1297	‐	‐	‐
Total skin (μm)	‐	‐		1458.7	‐	‐	‐
Skin capillitium							
Stratum corneum (μm)	‐	‐		‐	120.7	‐	
Total epidermis (μm)	‐	‐	24.7 ± 7.4 to 80.5 ± 3.0^a^	160.8	160.8	‐	‐
Dermis (μm)	‐	‐	777.5 ± 32.9 to 1143.1 ± 34.2^a^	1553.8	1714.6	‐	‐
Total skin (μm)	‐	‐		1714.6	1875.4	‐	‐
Skin back							
Stratum corneum (μm)	‐	‐		‐	‐	‐	‐
Total epidermis (μm)	‐	‐		150.3	‐	480 ± 153	650 ± 324
Dermis (μm)	4000^c^	‐		1330.6	‐	‐	‐
Total skin (μm)	‐	‐		1431.3	‐	‐	‐

a Minimum to maximum values Mean ± SE.

b Data corrected by the authors after publication.

c Thickest value.

Complete skin thickness measurement was found in studies 4 and 5: The thinnest skin value was 913.1 μm in the sole, whereas the thickest skin value was 1875.4 μm in the capillitium. Although these articles were from different publications, the main author and methods were the same in both studies.

Only studies 2 and 5 measured the thickness of the stratum corneum sub‐layer: Their values were 35.4 ± 11.03 μm in the abdomen region and 120.7 μm in the capillitium region. Total epidermis thickness presented a wide variation between studies 3, 4, and 5, even the biopsy site occurred in the same area of the body. Dimensions in the epidermis of capillitium ranged from 24.7 ± 7.4 μm in study 3‐160.8 μm in studies 4 and 5. In the sole, values ranged from 193.2 to 680 ± 315 μm in the reports 4 and 6 (newborns), respectively. As for the abdomen, the total epidermis thickness found was 161.6 μm in study 4, whereas in study 6, it was 650 ± 331 μm (newborns). On the skin over the back, total epidermis thickness was 150.3 μm in study 4 and 650 ± 324 μm (newborns) in study 6. Study 6 was the only one that evaluated the thickness of the fetal epidermis, where the sole measured 470 ± 121 μm, the abdomen 530 ± 111 μm, and the back 480 ± 153 μm.

Study 4 reported the thickness of the dermal layer as 719.9 μm in the sole. In the back region, studies 4 and 1 measured 1330.6 and 4000 μm, respectively. The dermal thickness of capillitium ranged from 777.5 ± 32.9 μm to 1714.6 μm, as reported by studies 3 and 5, respectively. The thickness of the dermal layer in the abdomen was 1297 μm, as reported by study 4.

## 4 DISCUSSION

The results of this review revealed lack of reliable evidence on histological sizes of skin thickness in newborns, supposedly golden‐standard values. The limited number of studies that met the eligibility criteria included the heterogeneity of methods of histological techniques of preparation and the different descriptions of layers/sub‐layers measures made difficult the summarization. Most articles analyzed morphometric and structural aspects of the skin using a qualitative approach. Among them, thickness measurement was the only one quantitative result, not always well described.

Smith et al^15^ provided an overview on the human dermal embryogenesis. In this study, more than one method of biopsy preparation and microscopy was used to describe skin development, as well as structural and biochemical properties. Fairley et al^16^ focused their analysis on the stratum corneum thickness of children with less than three months of age. In both studies, sizing process was performed with standard approaches of fixation, coloration, and metering with microscope. The authors described sampling collected during autopsy fixed in 10% buffered paraffin, and serially sectioned and stained with hematoxylin and eosin (HE), with measurements using microscope micrometric ocular filament, taking five measurements of each skin slide. De Viragh et al^17^ analyzed the parietal scalp thickness of seven newborns after two weeks of age. In this study, the sample preparation was different from the others, analyzing the depths perpendicular to the skin surface of five follicular segments, even with the same HE method of staining. In their morphometric analysis, the thickness of the epidermis and dermis was presented using a regression model along with aging, in years. For this, dataset considered mean skin depths from five follicular segments. Since both the epidermis and the dermis layers have a wave format, their minimum and maximum thicknesses were determined. We included in our review both boundary values, taking the expected numbers for zero days of life. The group Kakasheva‐Mazhenkovska et al^18^, ^19^ published two articles that included neonatal skin samples in 2011. The first analyzed skin samples from 15 distinct regions of the body,^18^ and the latter evaluated only structural components of the surface over capillitium.^19^ At least 12 skin biopsies per subject with 0.5 cm of size were included with total skin and part of the subcutaneous adipose tissue. They were histologically elaborated according to the standard paraffin technique. Morphometric analysis was done through the computational system for image processing and analysis (Lucia M, Version 3). Khalfa et al^10^ reported histological and cytological changes during fetal, embryonic, and neonatal development. For such, skin biopsies of 30 embryos and fetuses aged 2‐6 months and 15 newborns were used in this study. We extracted only sixth month fetuses and newborn data. The samples were fixed at optimum shear temperature and sectioned using a freezing cryostat at 5 μm and −24°C. In addition to histology, analyses were made by electron microscopy scanning and morphometry supported by the software ImageJ.

We did not perform a meta‐analysis since the studies reported different statistical descriptions for measurements on diverse anatomic sites of the body, layers, and sub‐layers. Therefore, the process of skin measurement did not allow comparability. Only three publications presented average with variability values of skin thickness dimensions and without statistical analysis of similarities and differences. Even so, there were no confidence interval values for measurements in any article. With incomplete descriptive or analytic statistics, comparisons taking mean measurements and variability were impossible. After data extraction, taking only size descriptions and average values without ranges or statistical rigor, the studies reported variations between thickness dimensions even in the same region of biopsy sampling. For instance, entire epidermis thickness in abdomen of neonates ranged from 150.3 μm (without range values) to 650 ± 324 μm, according to Kakasheva‐Mazhenkovska et al^18^ and Khalfa et al,^10^ respectively.

Five of the six studies provided neonatal skin data for reviewing and only two of the six reported fetal skin data. Considering that 5%‐18% of births occurred before 37 weeks of gestation,^20^ the evaluation of late‐fetal period in this review has clinical relevance. However, it was impossible to compare fetal and neonatal skin thickness. Skin is a dynamic tissue, engaged in a continuous process of keratinization of the epidermis and desquamation on the surface.^5^ Skin thickness has the potential to be a marker of the skin maturation as well as the structural architecture reported by Ersch et al^21^ Despite this, most scientific literature on the development of human skin has been derived from specialized books, which reported studies with a focus on morphological evolutionary description of the tissue. Little attention has been given to the thickness values of their layers or of the entire skin. A frequently cited study from 1980 by Holbrook et al^22^ affirmed that there is no regional variation in the epidermal layer during the first trimester of fetal life, with the exception of the foot, which was more advanced both in thickness and differentiation stages. During the second trimester, final keratinization over the epidermis occurred earlier in the head, foot, and hands according to Wolf.^23^

Such classical histological values, proportions, of layers and differences between the body sites of newborns did not corroborate with the characteristics obtained with in vivo, using noninvasive imaging approaches. Evaluating 436 skin ultrasonography images of 222 alive newborns with gestational age ranging from 24 to 41 weeks of gestation, Vitral et al^9^ reported mean epidermal thickness at the sole with similar dimension to the forearm. In addition, the median dermal thickness had a higher dimension at the sole than at the forearm and a negative correlation between the dermal layer thickness and gestational age. Noninvasive measurements have occupied space in tissue morphometry, making them closer to clinical practice challenges. Petersen et al^24^ associated the skinfold thickness of newborns with prematurity, analyzing ultrasonography echograms. Optical coherence tomography also proved to be a precise technique in terms of repeatability and reproducibility to measure skin layers.^12^ This is in part because they provide a view of in vivo tissue at real time, and also due to the dimensions being reliable and results more accurate than histological preparations. Another advantage over histological techniques is keeping the original tissue morphology.^9^, ^25^ However, invasive methods are still essential in situations such as diagnosis of skin‐related conditions,^26^, ^27^ as well as prenatal diagnosis of hereditary skin disease.^22^

The major limitation of this review was the low reproducibility of the findings extracted from primary articles, mostly without focus on the quantitative analysis of neonatal skin thickness. In addition, descriptions of techniques for metering were incomplete, which did not allow a proper comparison between studies and a mathematic summarization of results. In evidence‐based medicine, both measurement and standardization of measurement techniques are relevant in biomedical research.^1^ Without variability values or confidence intervals regarding layers and sub‐layers thickness dimensions being reported, there is no statistical evidence to confirm skin thickness variations between regions of the body as well as layers proportions of dermis and epidermis thickness, frequently reported in specialized text‐book reports regarding newborns’ skin.^28^

To date, a reliable histological thickness of newborns’ skin is unknown and there are only a few studies addressing this topic. This comprehensive review summarized evidence on skin thickness during birth obtained by invasive biopsy sampling of fetuses and newborns. Taking into consideration the importance of the skin barrier maturation in preterm neonates, for further investigations, analysis with better methodological quality will still be relevant to better sizing skin at birth, as well as correlating them to clinical challenges, such as maintaining temperature, infections, and other prognosis indicators.

## CONFLICTS OF INTEREST

Author ZSNR declares a patent deposit BR1020170235688 (CTIT‐PN862) approach to analyze newborn skin reflection and assess gestational age, on behalf of the Universidade Federal de Minas Gerais and Fundação de Amparo a Pesquisa de Minas Gerais, Brazil, http://www.fapemig.br/en/.

## AUTHORS CONTRIBUTION

IMFS and GLNV searched, interpreted, and analyzed the data, and wrote and revised the study. ZSNR designed this systematic review, interpreted and analyzed the data, and wrote and revised the study.
