# Supplementary material for: Skin thickness dimensions in histological section measurement during late‐fetal and neonatal developmental period: A systematic review
Source: Skin Res Technol. 2019 May 22;25(6):793–800. doi: 10.1111/srt.12719 (PMC6900000; doi:10.1111/srt.12719)
Supplement: Supplementary file 1 [file SRT-25-793-s001.docx]

**Supplementary file**

Table - Descriptive summary of the two full-text excluded articles

| Study | Fetal or neonatal specimen | Skin biopsy analysis | Human being | Measure the thickness of the skin or its layers |
| --- | --- | --- | --- | --- |
| Holbrook KA, Odland GF 1980^22^ | Y^a^ | Y^a^ | Y^a^ | N^a^ |
| Ersch J, stallmach T., 1999^21^ | Y^a^ | Y^a^ | Y^a^ | N^a^ |

^a^Y: yes

^b^N: no
